# Sequential Congruency Effects in Monolingual and Bilingual Adults: A Failure to Replicate Grundy et al. (2017)

**DOI:** 10.3389/fpsyg.2018.02476

**Published:** 2018-12-11

**Authors:** Samantha F. Goldsmith, J. Bruce Morton

**Affiliations:** Department of Psychology, Brain and Mind Institute, University of Western Ontario, London, ON, Canada

**Keywords:** cognitive control, Eriksen flanker task, bilingualism, bilingual advantage, sequential congruency effects

## Abstract

Previous research suggests bilingual adults show smaller sequential congruency effects than monolingual adults. Here we re-examined these findings by administering an Eriksen flanker task to monolingual and bilingual adults. The task produced robust conventional and sequential congruency effects. Neither effect differed for monolingual and bilingual adults. Results are discussed in terms of current debates concerning differences in cognitive control between monolingual and bilingual adults.

## Introduction

### Bilingualism and Cognitive Control: Are There Differences?

One longstanding and rather vexing question in the study of human psychology concerns whether a lifetime of bilingualism leads to measurable changes in cognitive control. Several accounts predict that it should. According to Green (1998), for example, everyday language use is challenging for bilinguals as it requires the selection of words and meanings from a target language amidst competition from translation equivalents of a non-target language. Because managing cross-language interference relies on general control processes, bilinguals become highly practiced—and thus advantaged—in problems of cognitive control relative to monolinguals.

### Mixed Evidence in Adults

Decades of research have yielded some empirical support for the bilingual advantage hypothesis, mostly in the form of evidence that the distracting effect of irrelevant stimuli is typically smaller for bilinguals than monolinguals (e.g., Bialystok et al., 2004). One aspect of the available evidence that is difficult to reconcile with a simple formulation of the bilingual advantage hypothesis is the fact that the bilingual advantage is more consistently observed in studies of monolingual and bilingual children than it is in studies of monolingual and bilingual adults. Several large-scale adult studies have failed to find any differences between monolinguals and bilinguals across a wide range of cognitive control tasks (Paap and Greenberg, 2013). And in cases where adult differences have been reported, these differences disappear after only a few blocks of trials (Bialystok et al., 2004). If the bilingual advantage reflects a lifetime of experience managing cross-language interference, why is the advantage more pronounced (not less) in young children than in adults? The growing number of large-scale replication failures has led a number of vocal critics to claim there is no coherent evidence for a bilingual advantage in cognitive control.

In defense of the bilingual advantage hypothesis, some have dismissed concerns about the null effects of adult studies. One argument is that adult response times in cognitive control tasks are quite small (on average, ~500 ms), and therefore group differences need to be large for statistically significant differences to emerge. For children, response times are considerably larger, and therefore group differences are easier to detect (see Grundy et al., 2017, p. 43). This argument is obviously flawed, as it is the variance of two distributions, rather than the difference in their means, that determines whether or not a group difference will be statistically significant. Moreover, because response time *variability* is greater in children than in adults, it is typically *harder* to detect group differences in children, even when the absolute value of those differences is larger.

**Figure 1:**
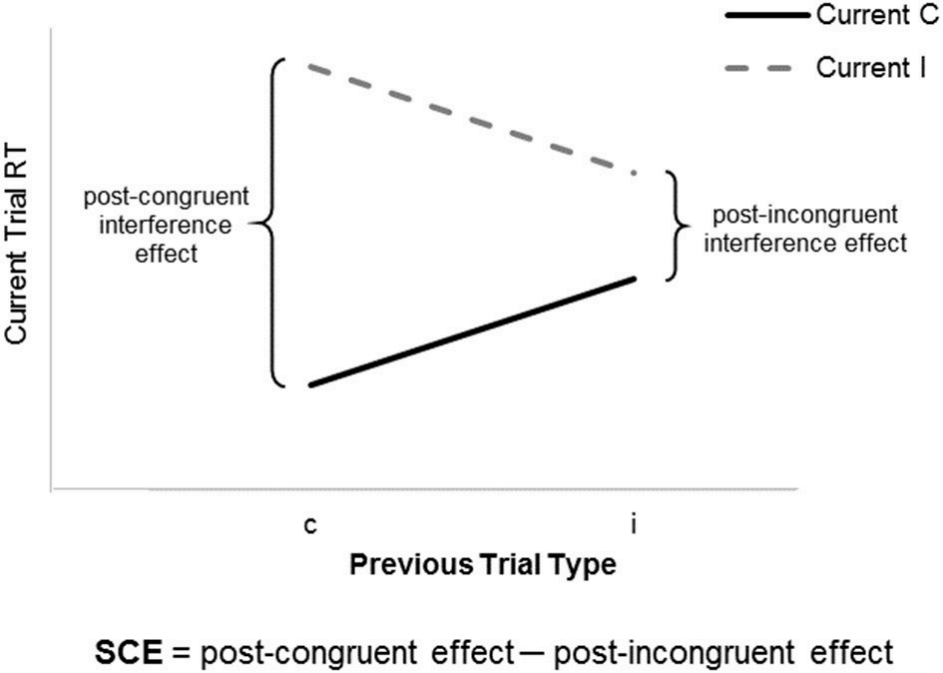
Depiction of the sequential congruency effect. Congruency effects, measured as the difference in RT across incongruent (I) and congruent (C) trials, are larger following previous congruent (c) than previous incongruent (i) trials. A single value for the sequential congruency effect is computed as the post-congruent interference effect (cI–cC) minus the post-incongruent interference effect (iI–iC).

A more interesting suggestion is that differences between monolingual and bilingual adults do exist, but are evident only given careful choice of cognitive control measures and analyses. Following this line of reasoning, Grundy et al. (2017) administered an Eriksen flanker task to groups of monolingual and bilingual adults. Across repeated trials, participants responded to the direction of a centrally presented arrow (press left key for “<”; press right key for “>”). On congruent trials, the target arrow was flanked by arrows pointing the same direction (< < < < < or > > > > >); on incongruent trials, the target arrow was flanked by arrows pointing the opposite direction (> > < > > or < < > < <). Groups were compared in two ways. First, they were compared in terms of a conventional congruency or interference effect, computed as the difference in response time on incongruent vs. congruent trials. Consistent with other findings (e.g., Paap and Greenberg, 2013), this conventional analysis revealed no difference between monolingual and bilingual adults. However, a second more advanced analysis compared groups in terms of a sequential congruency effect, computed as the difference in interference effects *following* congruent vs. incongruent trials (refer to Figure 1). Although relatively easy to estimate from flanker data, sequential congruency effects of monolingual and bilingual adults had not hitherto been compared. Interestingly, bilinguals showed a *smaller* sequential congruency effect than monolinguals: for bilinguals, interference effects measured after congruent trials were comparable to interference effects measured after incongruent trials, whereas for monolinguals, interference effects measured after congruent trials were larger than interference effects measured after incongruent trials. The findings provide a nice illustration of the idea that differences between monolingual and bilingual adults are subtle and may require careful choice of methods to reveal.

**Figure 2:**
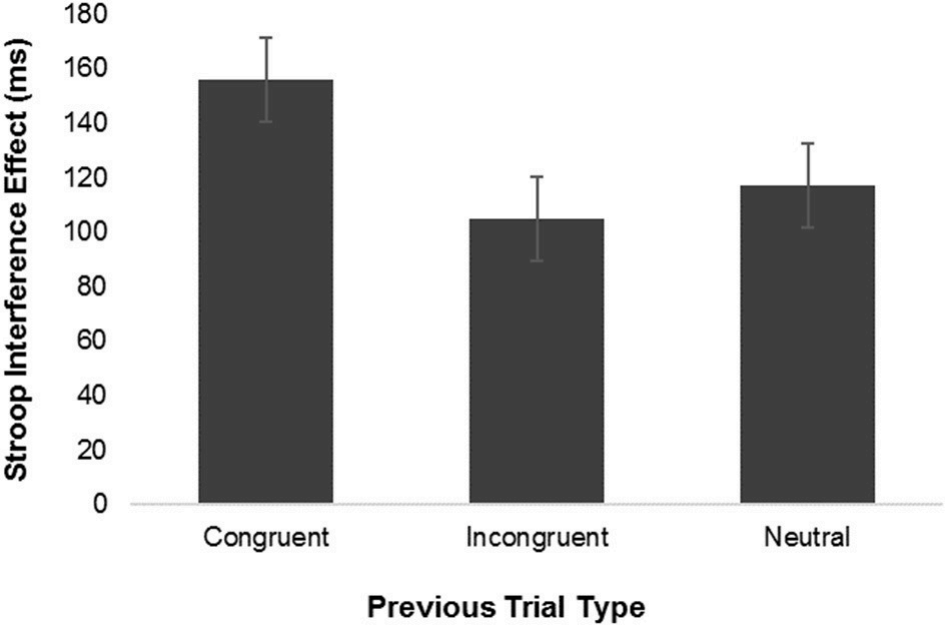
Results of Compton et al. (2012) showing that post-incongruent interference effects are comparable in magnitude to post-neutral interference effects, whereas post-congruent effects are greater than post-neutral. Findings suggest sequential congruency effects are driven more by adaptations to prior congruent than prior incongruent trials.

But what do these differences mean? According to Grundy et al., differences in the sequential congruency effect suggest that bilinguals more efficiently disengage attention from previous stimuli (both congruent and incongruent), affording them an advantage of greater attentional focus on current trials, relative to monolinguals. This claim is partially supported by evidence that greater practice on stimulus-response compatibility tasks is associated with smaller sequential congruency effects (e.g., van Steenbergen et al., 2015). That said, the claim that smaller conflict adaptation effects reflect some form of enhanced processing cuts against the grain of virtually every other model of sequential congruency effects. And while it is true that these alternative models are quite varied, there is at least a consensus among these accounts that the sequential congruency effect is fundamentally an expression of learning (for discussion, see Egner, 2014). The sequential congruency effect, after all, reflects an adaptation of current processing by prior experience. From this standpoint then, smaller sequential congruency effects for bilinguals than monolinguals point to a disadvantage in learning for bilinguals, and are difficult to reconcile with the view that bilinguals are advantaged in cognitive control (Green, 1998). Furthermore, contrary to various claims (Grundy et al., 2017; Bialystok and Grundy, 2018), evidence reported by Grundy et al. (2017) is equivocal on the issue of whether bilinguals show diminished influence of prior congruence, prior incongruence, or both, because there was no measurement of these effects relative to a neutral trial baseline. Prevailing models attribute the sequential congruency effect to an effect of prior conflict (e.g., Botvinick et al., 2001), but there is some evidence suggesting adaptation of current trial performance may be driven more by prior congruence than by prior incongruence (Compton et al., 2012; see Figure 2). Whatever the underlying basis of the sequential congruency effect, the fact that Grundy et al.'s data lacked a prior neutral trial baseline, it impossible to draw any conclusions about whether bilinguals show smaller adaptation effects following congruent trials, incongruent trials, or both.

### The Current Study

The present study therefore examined sequential congruency effects in monolingual and bilingual adults more closely, by comparing interference effects following congruent and incongruent trials with interference effects following neutral baseline trials. There were three alternative predictions. First, if bilingualism is associated with an advantage in learning and cognitive control (Green, 1998), bilingual adults should show a larger sequential congruency effect than monolingual adults, with effects being driven by prior congruence, prior incongruence, or both. Second, if bilingualism is associated with a *disadvantage* in learning and cognitive control (Grundy et al., 2017), bilingual adults should show a smaller sequential congruency effect than monolingual adults. Finally, if bilingualism is unrelated to learning and cognitive control (Paap and Greenberg, 2013), there should be no difference in the magnitude of the sequential congruency effect for monolingual or bilingual adults.

## Methods

### Participants

Seventy-three undergraduate students were recruited from Western University to participate in the study in exchange for course credit. Of these, 65 participants (26 males; mean age = 19.1 years, *SD* = 2.526) were included in the final sample. Data from seven participants were excluded owing to lower than 80% accuracy on the flanker task. Forty-four participants were bilingual (i.e., self-reported as fluent in at least two languages) and 21 were monolingual. Twenty-one bilinguals reported English as their first language, with others reporting Arabic, Chinese, Farsi, Korean, and Vietnamese. Nineteen monolinguals reported English as their first language and two reported Chinese.

### Measures

#### Demographic Questionnaire

Participants completed an eight-item demographic questionnaire that solicited information about participant age, gender, household income, parental education, and parental occupation.

#### Daily Language Use Questionnaire

Following procedures used elsewhere for assessing bilingual vs. monolingual language status (e.g., Grundy et al., 2017), participants completed a 7-item questionnaire that solicited information about participant first language, knowledge of other languages (if any), and typical day-to-day language use. Participants indicated the language(s) they typically use with family and friends, at school, when engaging with media, and when performing mental math. Responses to these items were selected from five options: “Only my first language,” “Mostly my first language,” “Both my first and other language(s),” “Mostly my other language(s),” and “Only my other language(s).”

#### Non-verbal Intelligence

Participants completed five computer-based measures of non-verbal intelligence including a forward digit span task, two spatial memory tasks, a pattern comparison task, and a mental rotation task.

#### Flanker Task (Eriksen and Eriksen, 1974)

The primary task was an Eriksen flanker task implemented in Python. Trials began with a white fixation cross centered on a black screen for 1,000 ms, followed immediately by a target stimulus embedded in flankers. On congruent trials, flankers pointed in the same direction as the target; on incongruent trials, flankers pointed in the opposite direction of the target; and on neutral trials, flankers consisted of two non-directional horizontal dashes. Stimuli were presented in the center of the screen for 1,500 ms or until a response was made. Participants were instructed to indicate as quickly and accurately as possible the direction the target stimulus. Participants responded by pressing the left- or right-most button on a five-button response box. To ensure response time was measured with the highest possible fidelity, we employed a Chronos button-box (Psychology Software Tools®) with sub-millisecond temporal resolution. The entire task consisted of 420 trials divided into four equal blocks. Participants completed the task in two two-block segments.

### Procedure

All procedures were reviewed and approved by the Western University Research Ethics Board. Participants were provided with a letter of information concerning the study and provided signed written consent to their participation.

All measures were completed on a desktop computer with a 15-inch color monitor. A research assistant remained in the testing room throughout testing to oversee the protocol administration. After providing consent, participants completed the demographic and language questionnaires. Participants then completed two 120-trial blocks of the flanker task, the five computer-based measures of non-verbal intelligence, and then two final 120-trial blocks of the flanker task. Testing took on average 45 min to complete.

## Results

### Demographics and Language Status

Most participants came from middle- or upper-class socioeconomic backgrounds with university-educated parents. Monolingual and bilingual participants had comparable socioeconomic backgrounds. Monolingual participants reported proficiency in only one language; bilingual participants reported balanced daily use of both languages (refer to Supplementary Table 1).

### Non-verbal Intelligence

Individual scores on each of the five non-verbal intelligence tasks were transformed into *z*-scores and summed to create an aggregate non-verbal intelligence score for each participant. Results of an independent samples *t*-test revealed no significant difference between aggregate scores of monolinguals (*M* = 0.542, *SD* = 2.438) and bilinguals (*M* = −0.259, *SD* = 3.036), *t*_(63)_ = 1.056, *p* = 0.295.

### Eriksen Flanker Task and Sequential Congruency Effects

Response times across all flanker trial types are presented in Table 1 separately for monolingual and bilingual participants. Response times were submitted to a 3-way mixed Analysis of Variance (ANOVA) with Current Trial (congruent, incongruent) and Previous Trial (congruent, incongruent) as within-subjects factors, and Group (monolingual, bilingual) as a between-subjects factor. There was an overall effect of Current Trial, *F*_(1, 63)_ = 351.5, *p* < 0.001, with response times on incongruent trials (*M* = 497.4 ms, *SD* = 47.8) significantly slower than response times on congruent trials (*M* = 423.9 ms, *SD* = 48.4). Current Trial congruency interacted with Previous Trial congruency, as reflected in a significant 2-way Current Trial × Previous Trial interaction, *F*_(1, 63)_ = 14.6, *p* < 0.001. This interaction reflects a sequential congruency effect and was driven by fact that Current Trial interference effects were greater following congruent trials (*M* = 81.1 ms; *SD* = 35.9) than following incongruent trials (*M* = 61.0 ms; *SD* = 31.3). No other effects or interactions were significant.

**Table 1:** Mean response times (ms) and associated standard deviations for sequential flanker pairs in monolinguals vs. bilinguals.

**Prior trial**	**Current trial**	**Language status**	***M***	***SD***
Congruent	Congruent	Monolingual	404.596	43.364
		Bilingual	420.700	49.057
	Incongruent	Monolingual	481.998	44.853
		Bilingual	503.616	56.978
Incongruent	Congruent	Monolingual	419.919	43.864
		Bilingual	432.725	57.656
	Incongruent	Monolingual	483.197	41.907
		Bilingual	492.619	48.862
Neutral	Congruent	Monolingual	420.578	43.060
		Bilingual	426.600	50.903
	Incongruent	Monolingual	491.115	42.322
		Bilingual	505.066	52.893

### Comparison of Post-congruent and Post-incongruent Interference Effects

**Figure 3:**
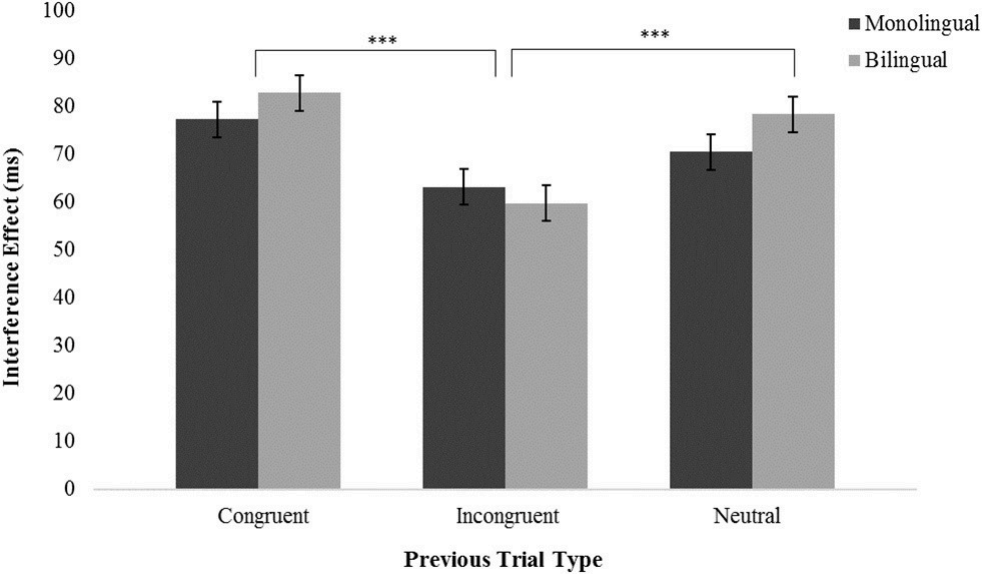
Post-incongruent interference effects were smaller than both post-congruent and post-neutral interference effects. There was no effect of Group and no interaction. ***p < 0.001.

To examine whether sequential congruency effects are driven more by prior congruent or prior incongruent trials and whether these effects differ for monolinguals and bilinguals, we compared post-congruent and post-incongruent interference effects with a post-neutral trial baseline, shown separately for monolinguals and bilinguals in Figure 3. A 2-way mixed ANOVA with Previous Trial (congruent, neutral, incongruent) as a within-subjects factor and Group (monolingual, bilingual) as a between-subjects factor, revealed an effect of Previous Trial on the current trial interference effect, *F*_(2, 63)_ = 17.2, *p* < 0.001, but no effect of Group and no Previous Trial × Group interaction. *Post-hoc* analyses indicated that current trial interference effects were smaller following incongruent compared to congruent trials (*M*_*D*_ = 20.1 ms, *p* < 0.001) and smaller following incongruent compared to neutral trials (*M*_*D*_ = 14.9 ms, *p* < 0.001). Current trial interference effects following previous congruent trials were not different than interference effects following previous neutral trials. No other effects or interactions were significant.

## Discussion

Monolingual and bilingual adults were administered an Eriksen flanker task. Participants exhibited a conventional congruency effect, as reflected by slower responses on incongruent compared to congruent trials, and a sequential congruency effect, as reflected by a larger congruency effect following congruent than following incongruent trials. There were however no differences in either conventional or sequential congruency effects of monolingual and bilingual adults.

The present findings contrast with evidence suggesting sequential congruency effects differ for bilingual and monolingual adults. Examination of sequential congruency effects have drawn some attention of late given mounting evidence that conventional measures of cognitive control fail to reveal differences between monolingual and bilingual adults (Paap and Greenberg, 2013). One recent study, for example, reported *smaller* sequential congruency effects for bilingual compared to monolingual adults (Grundy et al., 2017). According to received models of the sequential congruency effect (see Egner, 2014), such group differences point to a possible learning disadvantage for bilingual vs. monolingual adults. Others, however, have interpreted smaller sequential congruency effects for bilinguals as evidence that bilinguals disengage attention from congruent and incongruent stimuli more effectively than monolinguals (Bialystok and Grundy, 2018). We tested this idea directly by measuring interference effects following congruent and incongruent trials relative to a post-neutral trial baseline. Consistent with conflict-adaptation models of the sequential congruency effect (e.g., Gratton et al., 1992; Botvinick et al., 2001; but see Compton et al., 2012), adaptation of conflict processing in the current trial was influenced more by prior incongruent trials than by prior congruent trials. That said, we found no difference in the size of sequential adaptation effects of any kind—post-incongruent or post-congruent—evidenced by monolingual vs. bilingual adults. As such, our findings are inconsistent with the view that relative to monolinguals, bilinguals more effectively disengage attention from previous stimuli or exhibit disadvantages in learning. Instead, the present findings are most consistent with the idea that monolingual and bilingual adults are indistinguishable in terms of sequential adaptation specifically and cognitive control more broadly (Paap and Greenberg, 2013).

Of course, the present study has several important limitations. One critical limitation is that there was very little in the present data that allows us to even speculate why we found no differences between monolinguals and bilinguals whereas other groups have (e.g., Grundy et al., 2017). Comparisons of monolingual and bilingual adults are always challenging because group differences in language status typically encompass differences in other factors, such as socio-economic status, immigration status, and culture, that confound the basic influence of language status. Indeed, controlling for these factors has been shown to attenuate differences between monolingual and bilinguals, at least in studies of children (see Morton and Harper, 2007). In the present case, it is unclear whether cross-study differences in sample composition could explain differences in findings, as only basic demographic variables were measured. Similarly, we only used very rudimentary survey-based measures of daily language use to assess language status. Although these methods remain well-utilized in studies of monolinguals and bilinguals (see Grundy et al., 2017 as an example), they are ill-equipped to identify subtle differences between monolinguals and bilinguals or differences between different sorts of bilinguals (for discussion, see Baum and Titone, 2014). Clearly, advancing our understanding of language status effects on cognitive control will require adherence to higher methodological standards (for discussion, see Morton, 2015).

As a final note, our findings pertain only to possible differences between monolingual and bilingual adults. Identifying differences in adult samples has been a key challenge in bilingual advantage research and is what motivated Grundy et al. to examine sequential congruency effects more closely in the first place. Although recent large-scale studies of children also present negative evidence for the bilingual advantage hypothesis (see Dick, 2018), research in this area should remain a high priority given the wealth of previously published positive evidence and its enormous influence on the field.

## Ethics Statement

Western University Non-Medical Research Ethics Board. Participants provided written voluntary consent to their participation.

## Author Contributions

SG and JBM designed the study and wrote the manuscript. SG collected and analyzed the data.

### Conflict of Interest Statement

The authors declare that the research was conducted in the absence of any commercial or financial relationships that could be construed as a potential conflict of interest.
