# Supplementary material for: Sequential Congruency Effects in Monolingual and Bilingual Adults: A Failure to Replicate Grundy et al. (2017)
Source: Front Psychol. 2018 Dec 11;9:2476. doi: 10.3389/fpsyg.2018.02476 (PMC6297870; doi:10.3389/fpsyg.2018.02476)
Supplement: Supplementary file 1 [file Data_Sheet_1.pdf]

Table 1.

*Frequency statistics for daily language use by monolinguals (*n *= 21) and bilinguals (*n *= 44) across various contexts.*

| | | **Only 1st language** | **Mostly 1st language** | **Both 1st and other language(s)** | **Mostly other language(s)** | **Only other language(s)** |
|---|---|---|---|---|---|---|
| **Family** | Monolingual | 19 | 2 | 0 | 0 | 0 |
| | Bilingual | 9 | 10 | 17 | 7 | 1 |
| **Friends** | Monolingual | 19 | 2 | 0 | 0 | 0 |
| | Bilingual | 19 | 10 | 7 | 7 | 1 |
| **At School** | Monolingual | 17 | 2 | 2 | 0 | 0 |
| | Bilingual | 23 | 4 | 6 | 5 | 6 |
| **Media**[a] | Monolingual | 14 | 6 | 1 | 0 | 0 |
| | Bilingual | 15 | 8 | 17 | 1 | 3 |
| **Counting**[b] | Monolingual | 19 | 1 | 1 | 0 | 0 |
| | Bilingual | 24 | 8 | 5 | 5 | 2 |

[a]When engaging with media, including TV, internet, video games, etc.
[b]When completing mental counting or arithmetic
